# Identification of RNF150 as the hub gene associated with microsatellite instability in gastric cancer

**DOI:** 10.1038/s41598-023-39255-7

**Published:** 2023-08-01

**Authors:** Jun Pan, Qingzhi Lan, Shengbao Li

**Affiliations:** 1 Department of Gastroenterology, Taihe Hospital, Hubei University of Medicine, Shiyan, 442000 Hubei China; 2 Department of Pathology, Renmin Hospital, Wuhan University, Wuhan, 430060 Hubei China

**Keywords:** Cancer, Oncology

## Abstract

Gastric cancer (GC) is a common digestive tract malignancy with the sixth global incidence and third cancer-related deaths, respectively. Microsatellite instability (MSI), accounting for one of the molecular subtypes of GC, plays an important role in GC and is affected by a sophisticated network of gene interactions. In this study, we aimed to explore the expression pattern and clinical performance of MSI related gene in GC patients. Weighted gene co-expression network analysis (WGCNA) was exploited to single out the vital module and core genes in TCGA database. We applied the protein–protein interaction (PPI) and survival analysis to propose and confirm RNF150 as the hub gene in GC. Finally, we utilized immunohistochemistry (IHC) and reverse transcription-polymerase chain reaction (RT-PCR) to explore the expression pattern of RNF150 in GC patients. With the highest weight correlation and standard correlation, RNF150 was selected as the hub gene for following validation. In validation, data obtained from the test sets showed a lower expression of RNF150 in MSI GC compared to microsatellite stability (MSS) GC. Moreover, survival analysis shows that MSI GC patients with a lower RNF150 expression level displayed the longer OS time. Compared to the expression in normal gastric tissues, the protein level of RNF150 was virtually up-regulated in ten cases of GC tissues. Furthermore, RNF150 protein level was decreased in MSI GC samples compared to MSS GC samples. When validated the mRNA expression with RT-PCR in fresh GC tissues, we also found the similar trend. RNF150 was identified as a novel MSI-related gene in GC. It is expected to be an auspicious prognostic biomarker for GC patients.

## Introduction

Gastric cancer (GC) is a typical digestive tract neoplasm with the sixth global incidence and third and cancer-related deaths, respectively^1^. Despite improvements in the comprehension of etiology and underlying molecular mechanisms, the burden caused by GC remains high in several area including China^2^. Although the treatment of GC has been greatly improved, survival rates for patients with advanced GC remain unsatisfactory ^3,4^. For this point, more researches related to GC are needed to improve prognosis of these patients.

GC is divided into four subtypes, including EBV positive, microsatellite instability (MSI), genomically stable (GS) and chromosomal instability. MSI is a genetic variation as a result of the inheritance and epigenetic inactivation of DNA mismatch repair genes, which is considered to be associated with the occurrence of cancer^5^. Although the MSI phenotype is presented in many types of tumors, it is most common in GC and colorectal cancer^6^. In the gastric tumorous tissues, MSI is mainly related to the methylation of MLH1 promoter CpG island, which leads to a decrease in the expression level of MLH1^7^. According to statistics, MSI was detected in 10–25% of GC patients, and the rest were microsatellite stability (MSS)^8^. A positive diagnosis of MSI often has clinically important implications in cancers, including determining prognosis, risk assessment of familial cancer risks, and identifying cancer-prone individuals^9^. MSI tumors may have similar cancer genetic pathways and result in similar clinical outcomes^10^. More importantly, MSI tumors are less sensitive to routine anti-cancer treatments^11^.

Researchers have widely used bioinformatics methods to analyze microarray data for the prediction of key genes in various diseases^12^. Weighted gene co-expression network analysis (WGCNA) is an R package utilized for establishing co-expression gene structures, identifying key modules and hub genes in various diseases^13^. For instance, a recent research used WGCNA for the identification of LRRC26 and REP15 to be associated with long term outcomes of patients diagnosed with colorectal cancer^14^. Besides, another study demonstrated that NRP1 might serve as potential prognostic biomarkers for GC patients by WGCNA^15^. In this study, we applied WGCNA and PPI network analysis to discover novel biomarkers associated with MSI GC, and validated the expression pattern of MSI related gene using immunohistochemistry ( IHC) and RT-PCR.

## Methods

### GC datasets

Human GC mRNA expression data and clinical characteristics were obtained from the Cancer Genome Atlas (TCGA) (https://genome-cancer.ucsc.edu/) and Gene Expression Omnibus (GEO) database (www.ncbi.nlm.nih.gov/geo). TCGA-STAD, consisting of 121 MSI GC patients and 258 MSS patients, was defined as the training set. While GSE62254, consisting of 68 MSI GC patients and 232 MSS patients, was used as test set.

We used the R software^16^ and the R package named “Affy”^17^ for the preprocessing of raw data in the training set. The dataset undergoes a series of preprocessing such as RMA background correction, log2 transformation, quantile normalization, and the median-polish probe set summarization. Finally, data quality was checked by sample clustering.

### DEGs extracting

We divided the GC patients into two groups (MSI and MSS). The R package named ‘limma’ was exploited to identify DEGs between those two groups^18^. |log_2_ fold change (FC)|> 0.2 was decided as cutoff value to identify DEGs.

### Co-expression network construction

**Figure 1:**
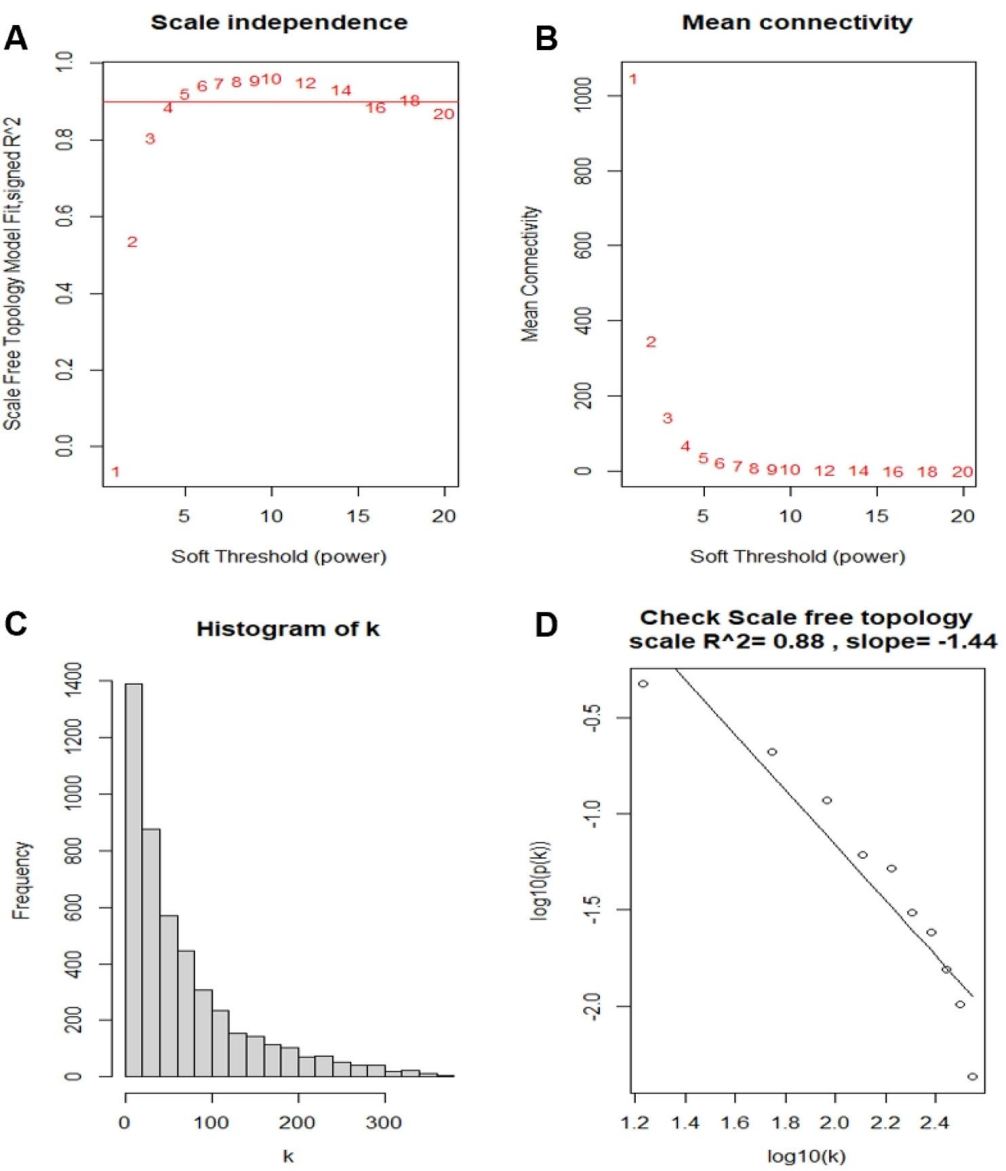
Calculation of soft threshold power in the WGCNA. (**A**). Analysis of the scale-free fit index for various soft threshold powers. (**B**). Analysis of the mean connectivity for various soft threshold powers. (**C**) Histogram of connectivity distribution (β = 4). (**D**) Checking the scale-free topology (β = 4). k: connectivity; p(k): possibility of the connectivity. *WGCNA* weighted gene co-expression network analysis.

Then we used those DEGs to construct co-expression structure using the ‘WGCNA’ package^19^. Firstly, we estimated the Pearson’s correlation matrices of paired genes. Secondly, we built up a weighted adjacency matrix by the power function a_mn_ =**|**cmn**|**^β^. The parameter β was a soft threshold, which could lay emphasis on solid correlations and weaken powerless correlations between genes. In our research, β = 4 (scale R^2^ = 0.88) is determined to establish a scale-free network (Fig. 1A–D). Then, the adjacency was adjusted into topological overlap matrix (TOM) that could estimate the network connectivity of every single gene^20^. Finally, based on the TOM-based measure of similarities and differences, we used the average linkage hierarchical clustering method to decide the minimum genome size of the gene dendrogram to be 30^21^.

### Identification of significant module

We applied two methods to determine important modules related with MSI. Linear regression analysis was performed between MSI status and various gene mRNA level. The *P* value was then log_10_ transformed into the gene significance (GS). Then we computed average gene significance for every single gene in the module to get the module significance. Next, we conducted principal component analysis, and took the major component as module eigengenes (ME). Finally, the key module was defined as with higher gene significance and higher correlation between module eigengenes and MSI.

### GO and KEGG analysis of DEGs

To examine the underlying mechanism of DEGs in the core module, we applied ‘clusterProfiler’ R package to conduct pathway analysis such as gene ontology (GO) and Kyoto Encyclopedia of Genes and Genomes (KEGG)^22^. Then, package ‘ggplot2’ was exploited to visualize the top ten pathways of GO and KEGG analysis. Finally, we exported genes in the key module and exploited the software Cytoscape to establish protein–protein interaction structure.

### Gene set enrichment analysis (GSEA)

In order to expound the underlying mechanism, we divided 121 MSI samples from TCGA database into low RNF150 group and high RNF150 group based on RNF150 mRNA level. Next, we used GSEA to explore functional pathway analysis and set the cutoff as *P* value < 0.05, gene size more than 30, and |enrichment score (ES)| more than 0.6.

### Hub gene validation

We performed survival analysis using the package named ‘survival’. We used test set GSE62254 to validate the expression pattern of the RNF150 between MSI and MSS patients. GraphPad Prism 8 was applied to analyze these data and visualize results. Statistical significance was estimated by two-tailed Student’s t-tests. *P* value less than 0.05 indicated statistical significance.

### Sample collection for validation

Ten GC patients from Renmin Hospital of Wuhan University during 2022 January and March were involved in this study. Paraffin Section tissues and their adjacent normal gastric mucosa were collected for IHC assay. Moreover, another 16 cases of GC from Renmin Hospital of Wuhan University during 2023 March and May were also recruited in this study and we prospectively collected the fresh tissues for RT-PCR assay. Our research was accepted by the Ethics Committee of Renmin Hospital of Wuhan University (No. WDRY2021-K002). All procedures are performed under the Declaration of Helsinki. All participants signed informed consent to allow their tissues to be used in this study.

### RT-PCR

RT-PCR was performed to determine the level of RNF150 mRNA in MSI and MSS GC tissues. Total RNA was extracted using Trizol (Invitrogen, USA) reagent. And using PrimeScript™ RT reagent Kit with gDNA Eraser (Perfect Real Time) (TaKaRa, Shiga, Japan; Cat No. RR047A) to synthesize cDNA. Real-time quantitative PCR was performed on a CFX Connect (BIO-RAD, USA) using TB Green Premix Ex Taq (TaKaRa; Cat. No. RR820A). The sense of RNF150 is GTAGAAGACATCGTG GCCATAAT, and the antisense of RNF150 is TCGAAACCTCTGGATGTAAT AAAAG. β-actin served as the internal reference gene (sense:GTCCACCGCAAATG CTTCTA, antisense:TGCTGTCACCTTCACCGTTC).

### Immunohistochemistry

IHC staining was conducted to explore the protein level of RNF150. Firstly, graded ethanol solutions were used to deparaffinize and rehydrate the tissue sections. Then, citrate buffer with concentration of 10 mM and pH of 6.0 were used for antigen retrieval of the sections. Subsequently, 3% H_2_O_2_ solution was used to block the activity of endogenous peroxidase. The tissue sections were incubated with RNF150 antibody (#HPA037987, Atlas Antibodies, USA). 3,3ʹ-diaminobenzidine (DAB) was used to develop the sections and hematoxylin was used to counterstained.

### Statistical analysis

All the statistical works were completed with SPSS software (version 18.0). Expression of RNF150 is divided into low expression and high expression based on the cutoff value. The prognostic significance of RNF150 mRNA in GC individuals was presented by Kaplan–Meier curves and determined by log rank test between the high and low groups of RNF150. ROC curves generated by R software (version 3.3) are utilized to determine the diagnostic power of RNF150 mRNA for differentiating MSI from MSS in GC samples. Pearson correlation was adopted in our analysis to determine the correlation between certain modules and MSI. *P* value less than 0.05 is regarded as statistically significant.

### Ethics approval

Our research was accepted by the Ethics Committee of Renmin Hospital of Wuhan University (No. WDRY2021-K002).

## Results

### DEGs extracting

**Figure 2:**
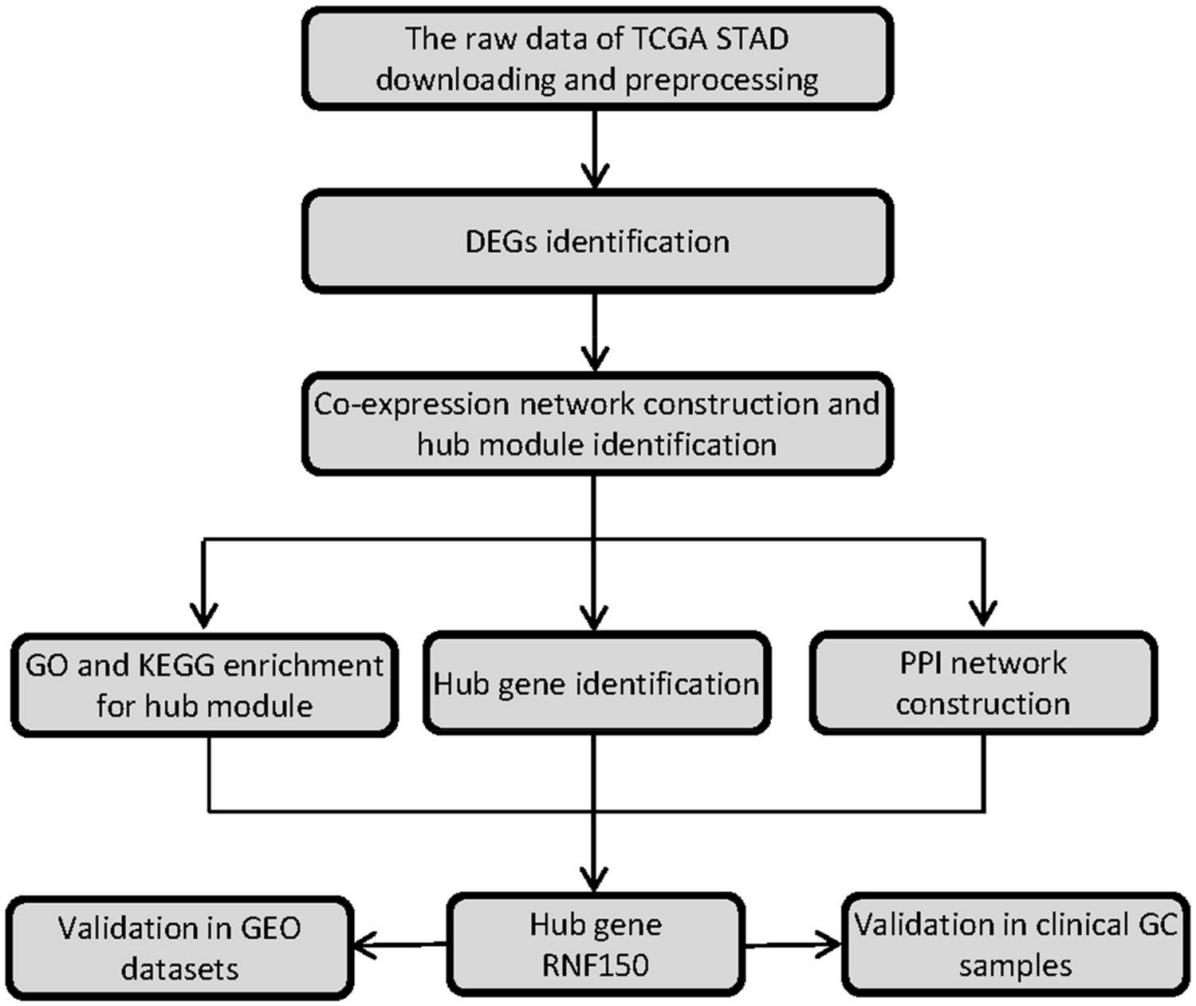
The flow diagram of this study.

Figure 2 displays the flow of this research. Using training set TCGA-STAD, we selected 4679 DEGs (2156 upregulated and 2523 downregulated in MSI samples) for following research with the cutoff of* P* value < 0.05 and **|**log_2_FC**|**> 0.2.

### Construction of co-expression network and key modules identification

**Figure 3:**
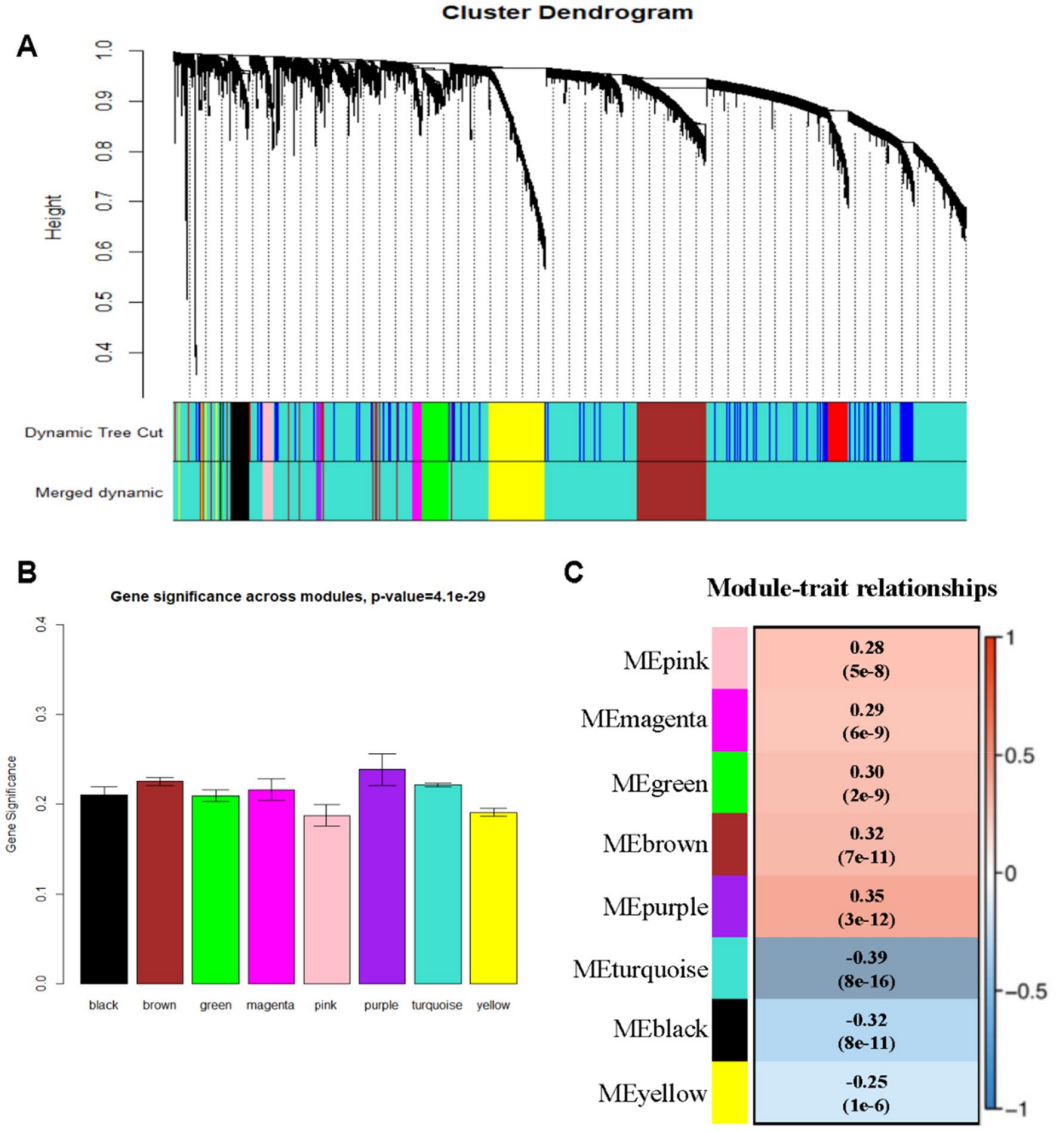
Identification of modules associated with MSI. (**A**) Dendrogram of all DEGs clustered based on a dissimilarity measure (1-TOM). (**B**) Distribution of average gene significance and errors in the modules associated with MSI in GC. (**C**) Heatmap of the correlation between module eigengenes and MSI. *GC* gastric cancer, *TOM* topological overlap matrix.

We exploited the package named ‘WGCNA’ to calculate 4679 DEGs into modules and 8 modules were visualized with various colors (Fig. 3A). Two approaches were used to select the core module related to MSI. First, these data indicated that the turquoise module had an upregulated MS value (Fig. 3B). Furthermore, the ME displayed that the turquoise module is the most significantly linked to MSI (r = −0.39, p = 8.07e−16) (Fig. 3C). Thus, the turquoise module was selected as the core module linked to MSI. Then we acquired the gene list from turquoise module for subsequent analysis.

For the reason of investigating the mechanism of the genes in GC we obtained from the turquoise module, we conducted pathway analysis. The top ten pathways in GO analysis were shown in Supplementary Fig. S1. Among biological processes, "regulation of cell morphogenesis" was the most significant enrichment, and "collagen-containing extracellular matrix" was the most significant enrichment in cellular components, and "cell adhesion molecule binding" was the most significant enrichment in molecular function (Supplementary Fig. S1A–C). Furthermore, our results show that proteoglycans in GC were the most significantly enrichment in the KEGG pathway analysis. (Supplementary Fig. S1D).

### Hub gene identification

**Figure 4:**
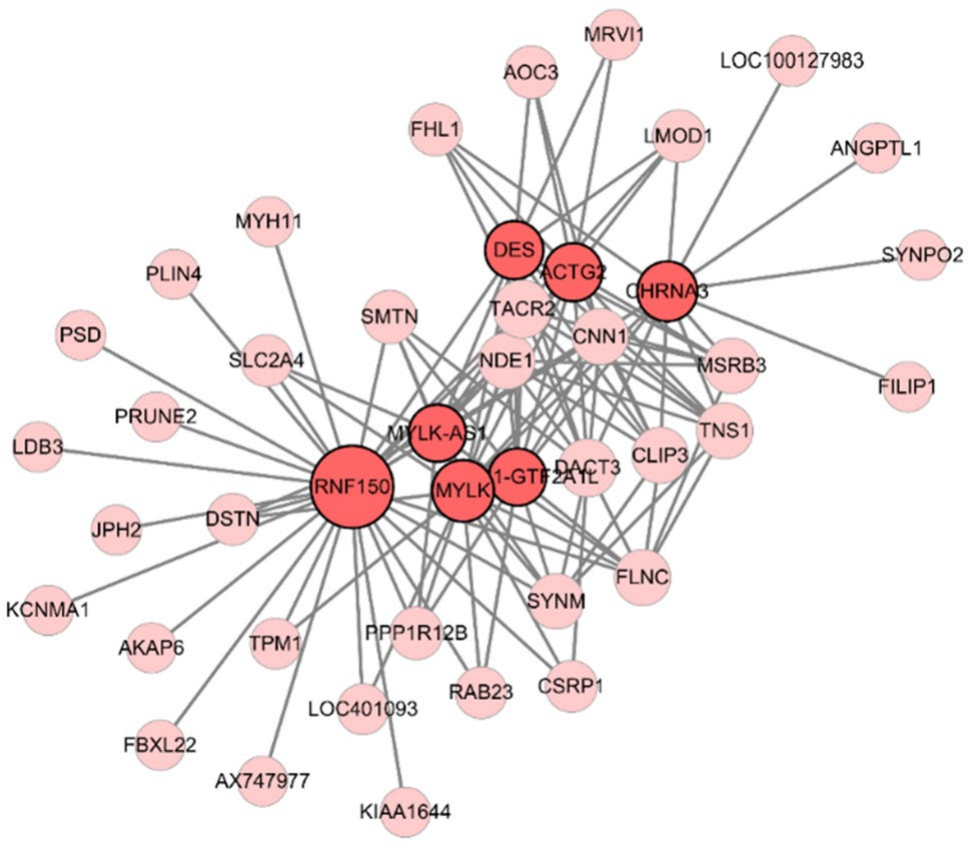
PPI network of genes in the turquoise module. Forty-three genes in the turquoise module were exported to construct a PPI network by Cytoscape. Each node size was proportional to the degree of connectivity in the weighted gene co-expression network.

Hub genes are defined as those who have closer connection with other genes in the same module. Among the turquoise module, we found the module membership (MM) of 43 genes were higher than 0.875. Then these 43 genes were chosen as hub gene candidates and exported to establish network of PPI using Cytoscape. Results show that 7 hub gene candidates were closely connected to other genes in the network (Fig. 4). Both with the highest weight correlation and standard correlation, RNF150 was finally defined as the core gene related to MSI GC.

### Hub gene validation

**Figure 5:**
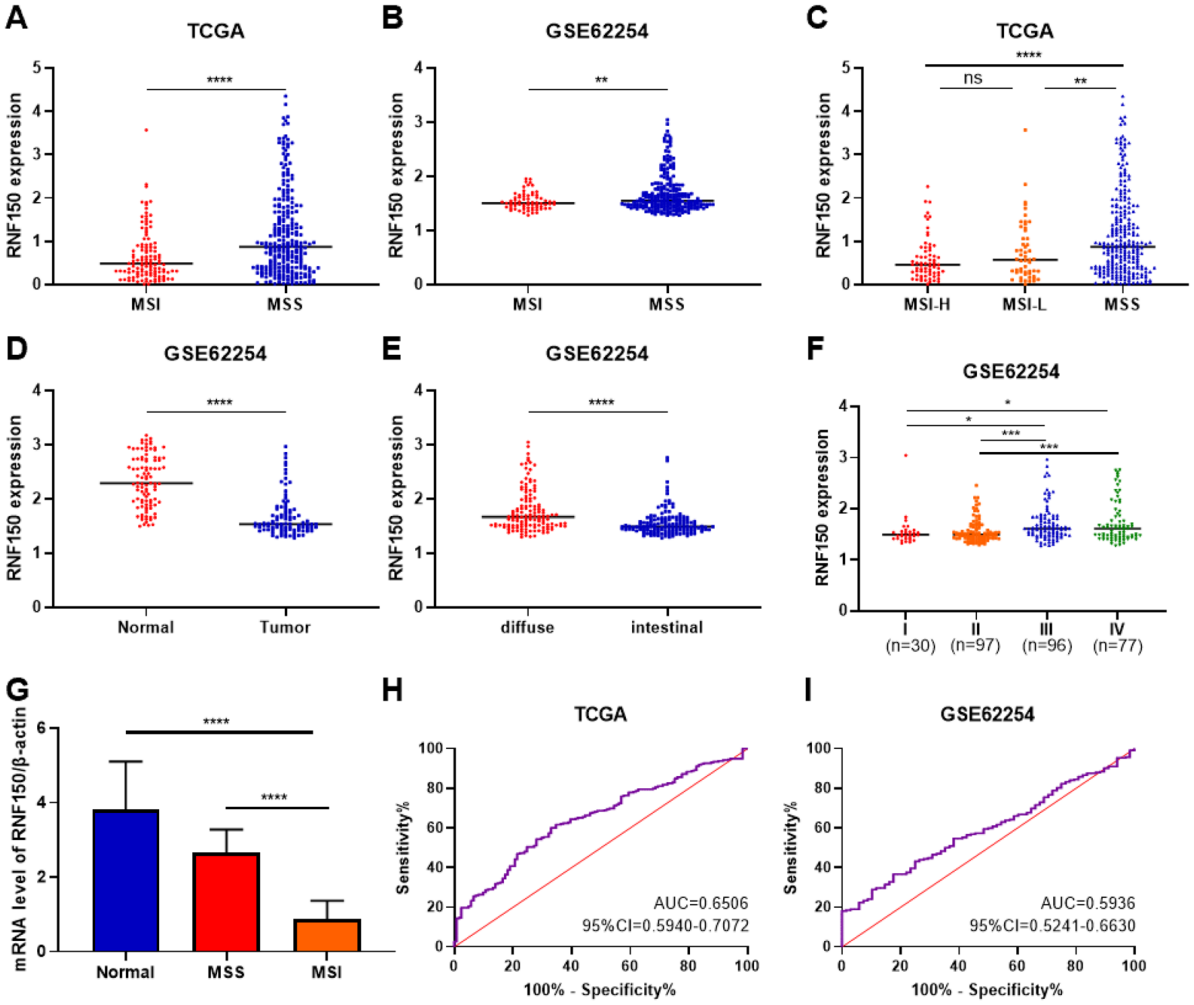
Hub gene validation. (**A**) RNF150 expression was downregulated in the MSI GCs compared with MSS GCs according to the TCGA database. (**B**) RNF150 expression was downregulated in the MSI GCs compared with MSS GCs according to the GSE62254 datasets. (**C**) The expression of RNF150 in patients with MSS, MSI-Low and MSI-High in the TCGA database. (**D**) RNF150 expression was downregulated in tumorous tissues compared with paired adjacent normal tissues in 98 patients in GSE62254. (**E**) RNF150 expression was downregulated in intestinal type tumorous tissues compared with diffuse type tumorous tissues in GSE62254. (**F**) RNF150 expression levels in the different TNM stage of GC patients in GSE62254. (**G**) RNF150 mRNA expression levels in clinical GC samples. Normal (n = 10), MSS (n = 6), MSI (n = 10). (**H**) ROC curve analysis of RNF150 mRNA levels in the TCGA database. (I). ROC curve analysis of RNF150 mRNA levels in the GSE62254. *p < 0.05, **p < 0.01, ***p < 0.001, ****p < 0.0001, *ns* not statistically significant, *TCGA* The Cancer Genome Atlas, *GCs* gastric cancers, *MSI* microsatellite instability, *MSS* microsatellite stability.

The RNF150 mRNA level was downregulated in MSI individuals compared to MSS individuals both in the TCGA-STAD and GSE62254 (Fig. 5A, B). According to the degree of MSI, GC was defined as MSI-High (MSI-H), MSI-Low (MSI-L) and MSS^23^. While according to the TCGA database, our results indicated that RNF150 mRNA level could definitely discriminate both MSI-H and MSI-L from MSS, but did not show obvious effect among MSI-H and MSI-L (Fig. 5C). In GSE62254, the RNF150 mRNA level was significantly up-regulated among paired adjacent normal tissues than that in tumor tissues in 98 patients (Fig. 5D). As for the Lauren classification of GC, the RNF150 mRNA level in diffuse-type was significantly downregulated compared to intestinal-type (Fig. 5E). Furthermore, according to their TNM stage, we categorized GC patients into stage I, stage II, stage III, stage IV. Results show RNF150 mRNA level increased with tumor development in patients with advanced tumor stage (Fig. 5F). Finally, we conducted ROC curve to explore the diagnostic value of RNF150 in distinguishing MSI from MSS GC patients. Results show that RNF150 exhibited a certain value both in TCGA and GSE62254 (Fig. 5H,I).

**Figure 6:**
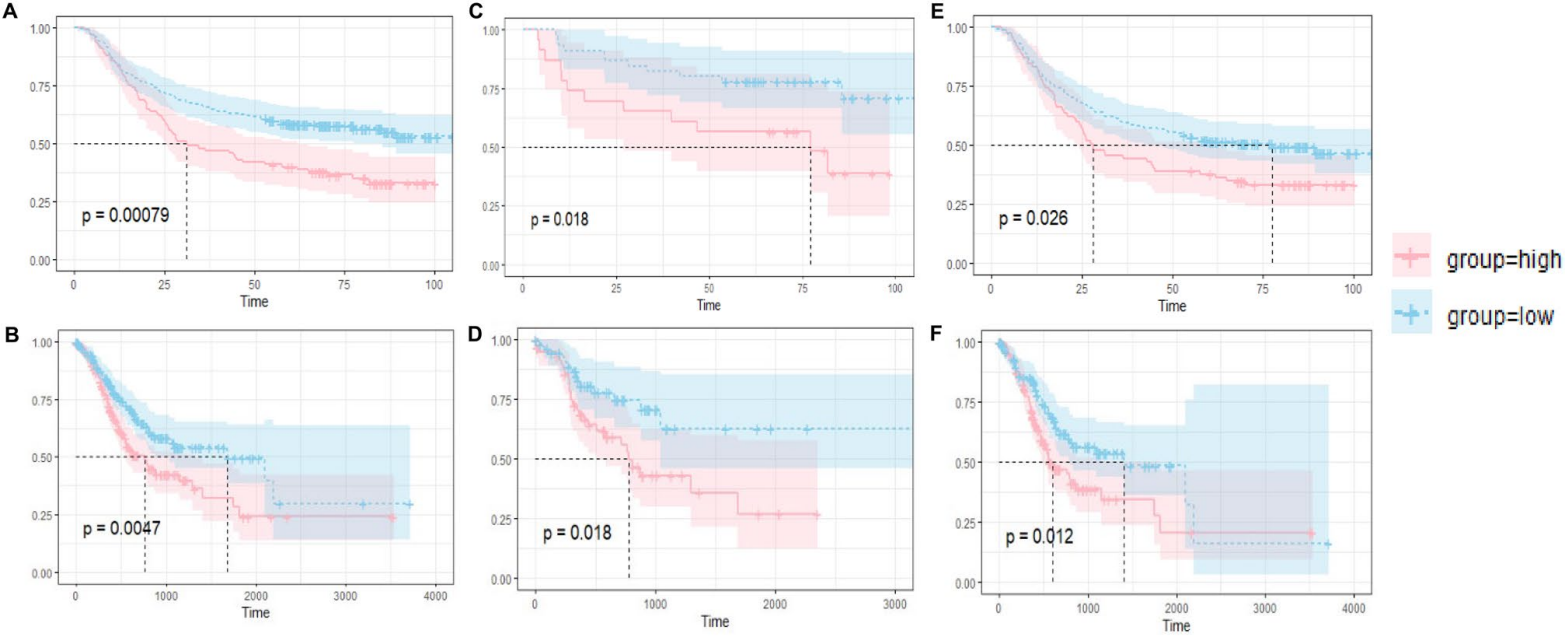
Hub gene validation. (**A**) Overall survival analysis of RNF150 with GC patients in GSEE62254. (**B**) Overall survival analysis of RNF150 with GC patients in TCGA database. (**C**) Overall survival analysis of RNF150 with MSI GC patients in GSEE62254. (**D**) Overall survival analysis of RNF150 with MSI GC patients in TCGA database. (**E**) Overall survival analysis of RNF150 with MSS GC patients in GSEE62254. (**F**) Overall survival analysis of RNF150 with MSS GC patients in TCGA database. *TCGA* The Cancer Genome Atlas, *GC* gastric cancer, *MSI* microsatellite instability, *MSS* microsatellite stability.

Furthermore, we analyzed the relationship between RNF150 mRNA level and the OS time of GC patient in both TCGA database and GSE62254. First of all, we separated all GC patients into two groups using cutoff value in TCGA and GSE62254, respectively. Results show that patients with a lower RNF150 expression level showed the longer OS time (Fig. 6A, B). Secondly, we separated MSI and MSS patients into two groups based on their RNF150 expression levels as mentioned above, and results remains the same trend (Fig. 6C–F).

### Gene set enrichment analysis

To determine the underlying biological mechanism of RNF150 associated with the KEGG pathway in MSI GC individuals, we conducted gene set enrichment analysis and found that “heparin binding” was enriched (Supplementary Fig. S2A). Besides, PPI network revealed that RNF150 may play an important role in the heparin binding pathway through the SLIT2 and RSPO3 gene (Supplementary Fig. S2B). Correlation analysis also showed that in MSI GC, the RSPO3 mRNA level was mostly related to RNF150 mRNA level (r = 0.8239, Supplementary Fig. S2C). We also analyzed the correlation between RNF150 and four MSI related genes (MLH1, MSH2, MSH6, PMS2) and results show that there is correlation between them (Supplementary Fig. S3).

### Validation in clinical GC samples

**Figure 7:**
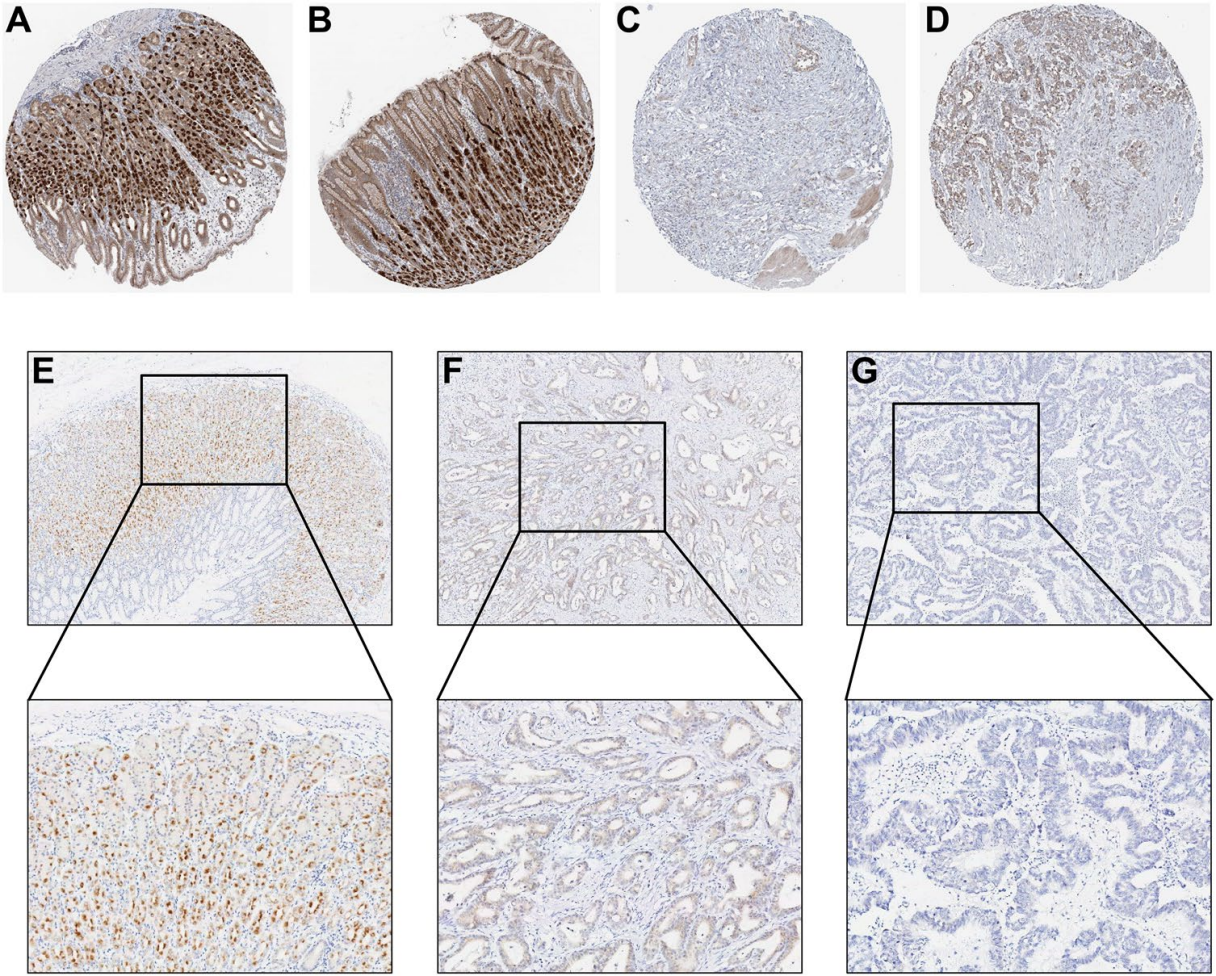
RNF150 protein level in GC samples. (**A,B**) Expression of RNF150 protein in normal gastric tissues from HPA database. (**C,D**) Expression of RNF150 protein in gastric cancer tissues from HPA database. (**E**) Expression of RNF150 protein in normal gastric tissues. (**F**) Expression of RNF150 protein in MSS GC tissues. (**G**) Expression of RNF150 protein in MSI GC tissues.

To further explore RNF150 protein level in GC samples, we applied the human protein atlas in our research. The results showed that the staining intensity of RNF150 in gastric tumors was significantly lower than that in normal tissue (Fig. 7A–D). For the sake of improving the reliability of our data, we acquired paraffin embedded section of 10 GC patients, in which five patients were MSS and five patients were MSI. We obtained tumor tissues (N = 10) and paired adjacent normal tissues (N = 10) simultaneously. Then we conducted IHC for RNF150 on tumor sections and paired adjacent normal sections from those GC patients. The representative images showed that the staining intensity of RNF150 in gastric tumors was significantly lower than that in normal tissues (Fig. 7E–G), and all the IHC images were shown in Supplementary Fig. S4. Furthermore, RT-PCR assay was utilized to verify the expression pattern of RNF150 mRNA in GC tissues and normal gastric tissues from Renmin cohort. As exhibited in Fig. 5G, expression of RNF150 mRNA in GC tissues (N = 16) was remarkably lower than that in normal gastric tissues (N = 10). RNF150 mRNA level was decreased in MSI individuals (N = 6) compared with MSS individuals (N = 10), which is in accordance with results we obtained in TCGA-STAD and GSE62254 datasets.

## Discussion

In the present study, we amied to identify hub genes involved in MSI in GC. By using TCGA database for WGCNA analysis, we identified the turquoise as the key module. Furthermore, we performed GO, KEGG and PPI analysis and discovered the RNF150 as the hub gene. Moreover, by cross validation with GEO datasets, we confirmed that mRNA levels of RNF150 were significantly down-regulated in GC than normal gastric mucosa, and low level of RNF150 mRNA predicted much better survival in GC patients.

RNF150 is a member of ring finger protein family. Eukaryotic cells contain a huge amount of RNF proteins, most of which function as E3 ubiquitin ligases^24^. E3 ubiquitin ligases, bind E2 to substrate and transfer ubiquitin molecules from E2 to substrates, is one of the three enzymes required to regulate protein ubiquitination. The other two enzymes are E1 ubiquitin-activating enzyme that hydrolyses ATP and E2 ubiquitin-conjugating enzyme that receives the ubiquitin from E1^25^. E3 ubiquitin ligases play a vital role in sustaining protein stability by ubiquitinating and degrading proteins misfolded^26^. Besides, E3 ubiquitin ligases also participated in other biological processes, such as cell proliferation, apoptosis, DNA damage repair, and intracellular vesicle trafficking, etc.^27^. Although there are few literature reports on RNF150, the role of many RING finger E3 ligases in malignant tumors is well known, including the oncogene MDM2^28^ and the suppressor gene BRCA1^29^. For instance, MDM2 can cause degradation of p53 through its E3 ligase activity^30^. According to reports, the MDM2/p53 pathway is associated with the development of GC^31^. In addition, BRCA1 can encourage DNA adjustment and simultaneously steady p53 through ubiquitination^32^. The study shows that the BRCA1 mRNA level is decreased in gastric cancer tissue and decided whether platinum-based chemotherapy had a response^33^.

The MSI GCs displayed distinct biological features from MSS GCs. Several studies have shown that MSI is associated with good overall survival of gastric cancer patients^34–38^. Karol el at found that stage I–III GC patients with MSI-H showed longer OS time in spite of positive status of margin^38^, while Stefania Beghelli and colleges declared that MSI in GC is linked to superior outcomes only with stage II patients^39^. In our study, we found that RNF150 is down-regulated in MSI GCs, while down-regulation of RNF150 predicts a longer survival of GC patients. Our results are in accordance with the previous study that MSI GC showed superior outcomes in GC patients. However, it’s too soon to get a conclusion about the exact role of MSI in GC patients. Two studies demonstrated that the detection of microsatellite instability has limited prognostic value in GC. Therefore, the underlying mechanism of MSI GC is still unclear and need further research to reveal.

Gene Set Enrichment Analysis (GSEA) does not require a definite threshold for differentially expressed genes. The algorithm analyzes the overall trend based on the actual situation, and from the perspective of gene set enrichment. It is easier to encompass the impact of subtle but synergistic changes on biological pathways. We used GSEA in our analysis and identified heparin binding is the most significant pathway in GC. Heparin binding plays an important role in acute stress, inflammation and tumor progression^40^. Heparin binding contributes to the up-regulation of heparanase, which associates with tumor vascularity and less favorable postoperative survival of cancer individuals^41^. Another study reveals that heparin binding promotes the angiogenic activity of tumor cells ^42^. Hence, the result of GSEA suggests that RNF150 might be involved in the progression of GC partly via heparin binding which correlates well with angiogenesis and hematogenous metastasis.

We need to mention limitations in this study. Firstly, studies on RNF150 are very limited so it’s difficult for us to hypothesize the potential mechanism of RNF150 affecting GC cells. Secondly, the prognostic role of RNF150 needs to be further explored in clinical individuals by different experimental methods such as western blotting and immunofluorescence. Finally, further cellular and animal experiments are needed in the future to expound the underlying mechanism of RNF150 in GC patients.

In brief, we recognized RNF150 as the vital gene linked to MSI in GC. Lower mRNA level of RNF150 predicts a longer survival of GC patients. Therefore, we propose that RNF150 is a novel biomarker and this study has important clinical implications for the development of new therapies for GC patients.

## Conclusion

Our study identified RNF150 as a novel biomarker in MSI GC. It is expected to be an auspicious prognostic biomarker for GC patients.

## Supplementary Information

Supplementary Figures.

## Data Availability

The datasets used and analyzed during the current study are available from the corresponding author on reasonable request.
