# Supplementary material for: Identification of RNF150 as the hub gene associated with microsatellite instability in gastric cancer
Source: Sci Rep. 2023 Aug 1;13:12495. doi: 10.1038/s41598-023-39255-7 (PMC10393951; doi:10.1038/s41598-023-39255-7)
Supplement: Supplementary file 1 — Supplementary Figures. [file 41598_2023_39255_MOESM1_ESM.docx]

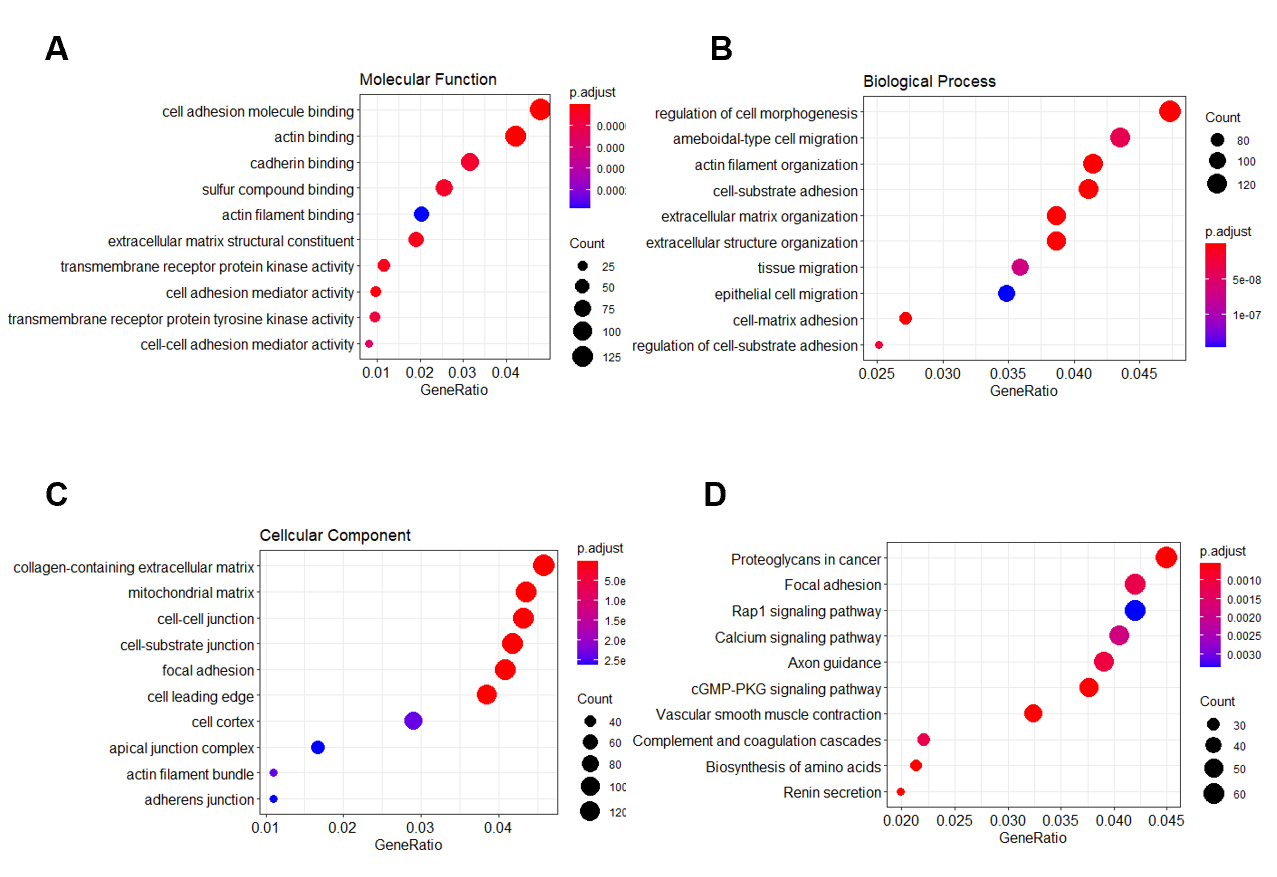


**Supplementary Figure 1. Functional enrichment analysis of all the genes in the turquoise module.**

(A). GO analysis for molecular function.

(B). GO analysis for biological process.

(C). GO analysis for cellular component.

(D). KEGG pathway enrichment analysis.


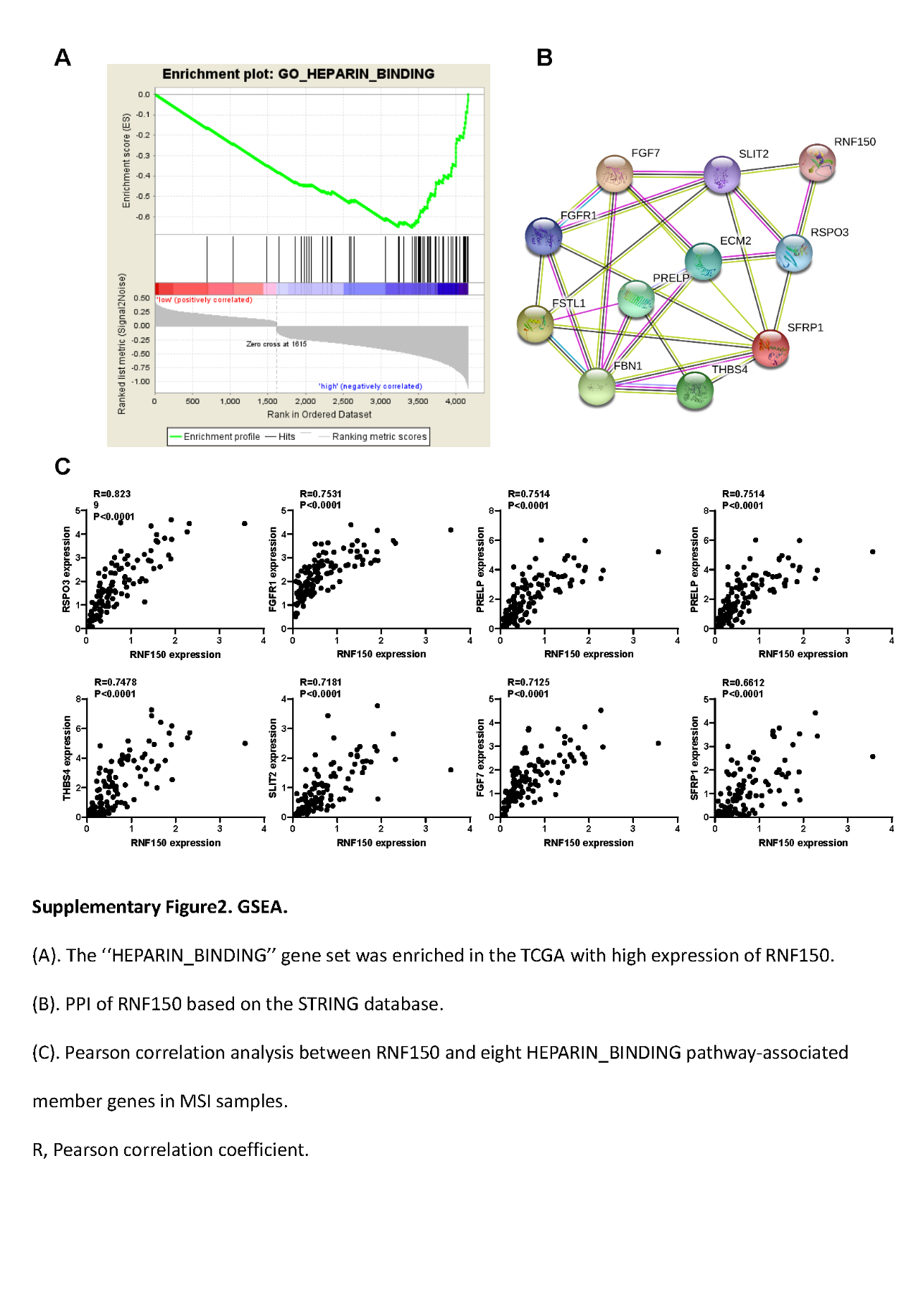


**Supplementary Figure2. GSEA.**

(A). The ‘‘HEPARIN_BINDING’’ gene set was enriched in the TCGA with high expression of RNF150.

(B). PPI of RNF150 based on the STRING database.

(C). Pearson correlation analysis between RNF150 and eight HEPARIN_BINDING pathway-associated member genes in MSI samples.
R, Pearson correlation coefficient.





**Supplementary Figure3.** Pearson correlation analysis between RNF150 and four MSI related genes in TCGA GC samples.
R, Pearson correlation coefficient.


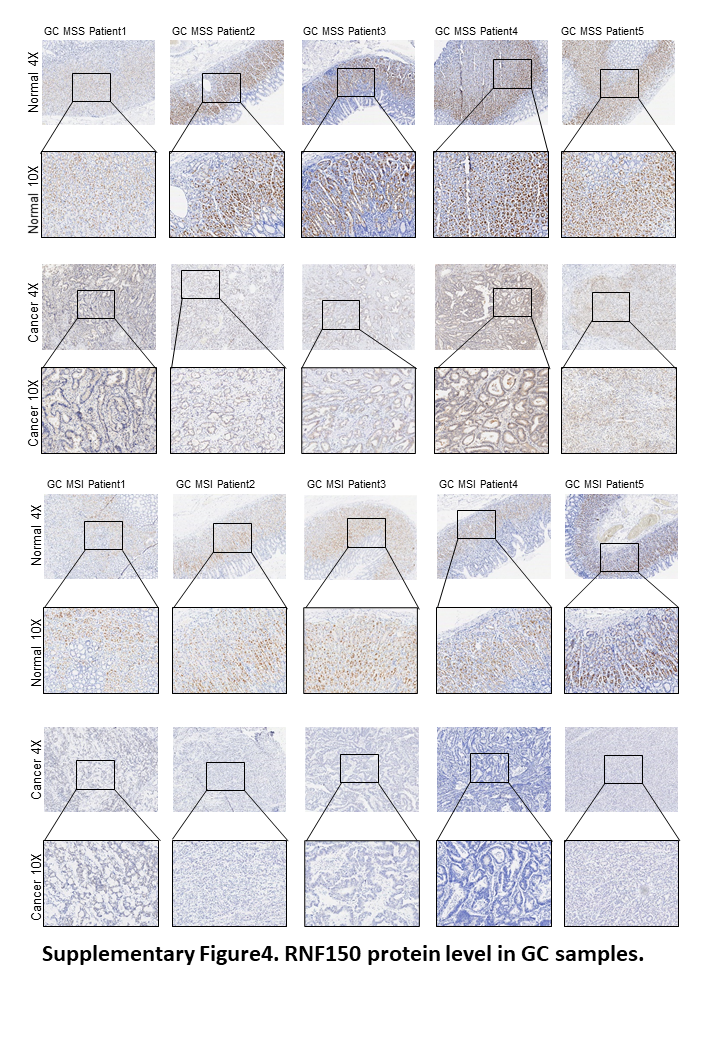


**Supplementary Figure4.** RNF150 protein level in GC samples.
